# Creutzfeldt–Jakob disease associated with a T188K homozygous mutation in the prion protein gene: a case report and review of the literature

**DOI:** 10.1080/19336896.2022.2031719

**Published:** 2022-02-07

**Authors:** Yuheng Shan, Jiatang Zhang, Yuying Cen, Xiaojiao Xu, Ruishu Tan, Jiahua Zhao, Shengyuan Yu

**Affiliations:** a Medical School of Chinese PLA, Beijing, P.R. China; b Department of Neurology, The First Medical Centre, Chinese PLA General Hospital, Beijing, P.R. China; c Department of Neurology, Characteristic Medical Centre of People’s Armed Police Force, Tianjin, P.R. China

**Keywords:** Creutzfeldt–Jakob disease, homozygote, prion protein gene, T188K

## Abstract

Genetic Creutzfeldt–Jakob disease (gCJD) is a prion disease caused by mutations in the prion protein gene (*PRNP*). It has an autosomal dominant inheritance, so gCJD with homozygous mutations is extremely rare, and the influence of homozygous mutations on the gCJD phenotype is unknown. We describe the clinical and laboratory features of a patient with a *PRNP* T188K homozygous mutation and perform a literature review of gCJD cases with *PRNP* homozygous mutations. The patient was presented with cerebellum symptoms, cognitive decline and visual disturbances. Auxiliary examinations revealed restricted diffusion in magnetic resonance imaging and glucose hypometabolism on ^18^Fluorodeoxyglucose-positron emission tomography. No periodic sharp wave complexes were detected in electroencephalography, and the cerebrospinal fluid 14-3-3 protein was negative. *PRNP* sequencing revealed the presence of a homozygous T188K variant. The patient died 15 months after disease onset. A literature review revealed *PRNP* V203I, E200K and E200D as the only three mutations reported as homozygous in gCJD. To the best of our knowledge, this is the first report of a gCJD patient with a *PRNP* T188K homozygous mutation. Although the clinical manifestations of our patient were similar to those with *PRNP* T188K heterozygous mutations, she presented with a slightly earlier onset and had a longer survival time. This is consistent with previous observations from patients with *PRNP* V203I and E200K homozygous mutations. Further studies are essential to clarify the influence of homozygous mutations on the gCJD phenotype.

## Introduction

Creutzfeldt–Jakob disease (CJD) is a rare and devastating neurodegenerative disorder pathologically characterized by the accumulation of abnormal prion protein (PrP^sc^), neuronal cell death and vacuolation in the central nervous system [1]. Approximately 85%–90% of CJD cases are sporadic, while about 10%–15% are genetic CJD (gCJD) caused by mutations in the prion protein gene (*PRNP*) [2]. Currently, more than 30 distinct genetic mutations, including point, deletion and insertion mutations, have been described in gCJD cases [3].

gCJD is inherited as an autosomal dominant trait and most patients are heterozygous for the mutations. Here, we describe a gCJD patient with a *PRNP* T188K homozygous mutation, which has not previously been reported as homozygous in gCJD. We also reviewed previous literature about homozygous mutations in PRNP as the clinical features of gCJD patients carrying these mutations are unclear because of their extremely low frequency.

## Patient and methods

### Clinical summary

**Figure 1.**
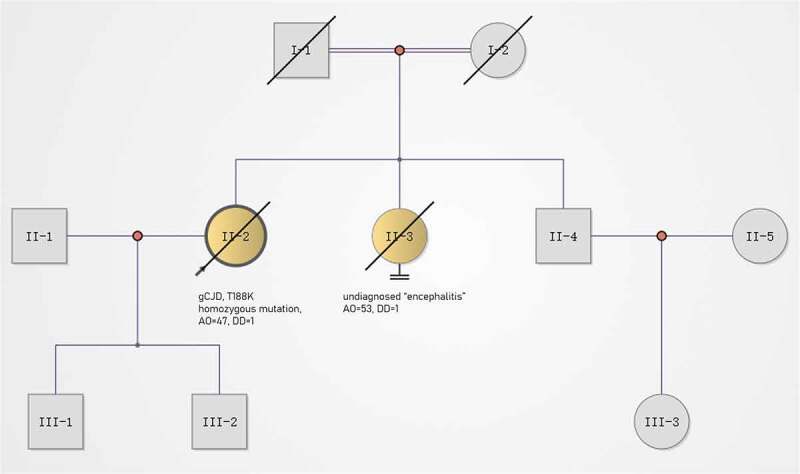
Pedigree of this case. gCJD, genetic Creutzfeldt–Jakob disease; AO, age of onset (year); DD, duration from onset to death (year). Circles indicate females; squares indicate males; yellow symbols indicate affected individuals; diagonal bars indicate deceased members; black arrow indicates the pro-band.

A 47-year-old woman with a 3-month history of headache and movement disorders was admitted to our hospital in December 2015. Her initial symptom was headache (at onset), presenting with diffuse and dull pain. One month after onset, the patient developed dizziness, diplopia and clumsiness of the left upper limb. Two weeks before admission, she was unable to walk steadily and her right upper limb also became clumsy. On admission, the patient developed cerebellar dysarthria but had no obvious cognitive impairment. Her past medical history was unremarkable. Her father died in his 50s from pancreatic cancer, and her sister died of undiagnosed ‘encephalitis’. Notably, her parents were close relatives (Figure 1).

**Figure 2.**
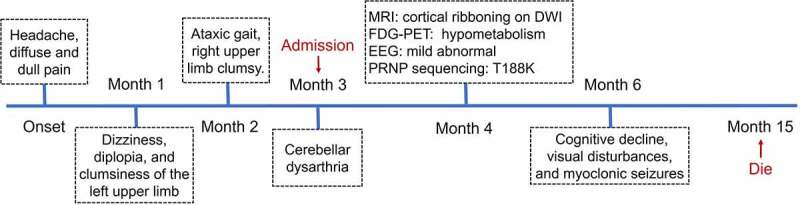
Timeline of the clinical manifestations and the results of examinations of this case. MRI, magnetic resonance imaging; DWI, diffusion-weighted imaging; FDG-PET, ^18^Fluorodeoxyglucose-positron emission tomography; EEG, electroencephalography.

**Figure 3.**
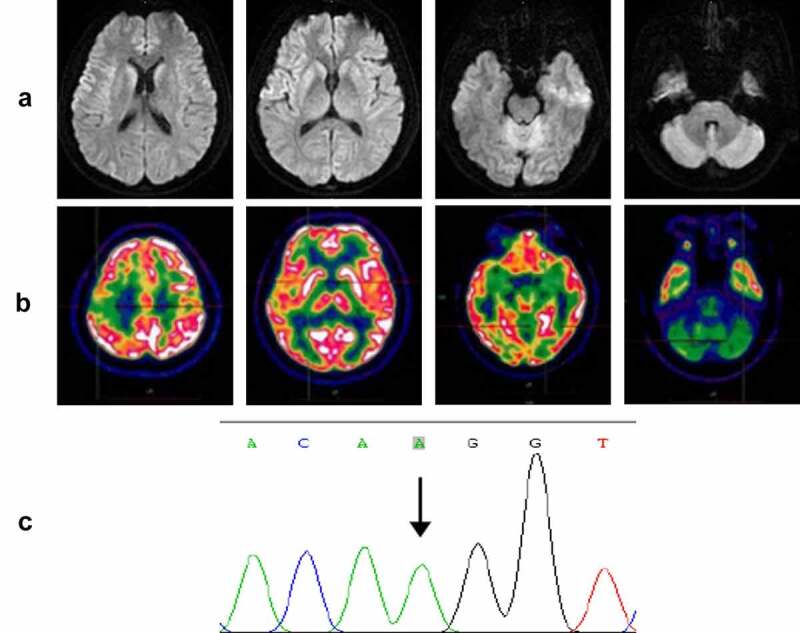
(a) Axial serial diffusion-weighted MRI showing restricted diffusion involving the right striatum, cerebellar cortex and cortical ribboning of the temporal and insular cortices; (b) ^18^Fluorodeoxyglucose-positron emission tomography showing severe glucose hypometabolism in the bilateral cerebellar cortex and mild glucose hypometabolism in the right frontoparietal cortex; (c) *PRNP* sequencing showing a homozygous substitution: c.563 C > A (p.T188K).

On admission, neurological examination revealed obvious diplopia, ataxia, left limb upper motor signs and left upper limb hypoesthesia, but no cognitive decline. A timeline of the clinical course is presented in Figure 2. Diffusion-weighted brain magnetic resonance imaging (MRI) (at 4 months after onset) showed restricted diffusion involving the right striatum, cerebellar cortex and cortical ribboning of the temporal and insular cortices (Figure 3a). ^18^Fluorodeoxyglucose-positron emission tomography (FDG-PET) (at 4 months after onset) revealed severe glucose hypometabolism in the bilateral cerebellar cortex and mild glucose hypometabolism in the right frontoparietal cortex (Figure 3b). Electroencephalography (EEG) was performed twice (at 4 and 5 months after onset), but no periodic sharp wave complexes (PSWCs) were detected. The patient’s cerebrospinal fluid (CSF) examination was unremarkable, and 14-3-3 protein was negative. *PRNP* sequencing identified a homozygous base substitution: c.563 C > A (p.T188K), coupled with homozygosity for methionine at codon 129 and glutamine at codon 219 (Figure 3c). Over the next few months, the patient continued to deteriorate, gradually developing cognitive decline, visual disturbances and myoclonic seizures. Her condition reached a state of akinetic mutism 12 months after onset. She eventually died 15 months after the onset of illness. Written informed consent from the patient and caregivers was obtained.

### Literature review

To give an overview of gCJD with homozygous *PRNP* mutations, we performed a literature review of both PubMed and Web of Science databases using the following keywords: (‘prion protein gene’ OR ‘PRNP’) AND ‘homozygote’. The literature review identified three articles reporting 12 gCJD cases with homozygous *PRNP* mutations (Table 1).

**Table 1.** Clinical and investigational features of patients with homozygous mutations in the *PRNP.*

Author, year, country	Age at onset (years), sex	Homozygote mutation	Codon 129	Initial clinical manifestation	Hyperintensity on DWI	PSWCs in EEG	CSF 14–3–3 protein	Disease duration, (months)
Komatsu et al, 2014, Japan	72, F	V203I	M/M	Cognitive dysfunction, gait disturbance	Basal ganglia, cerebral cortex	+	+	24
Nitsan et al, 2020, Israel	40, M	E200K	NA	Behavioural changes, 40%; cognitive decline, 30%; focal neurologic deficits, 20%; cerebellar symptoms, 0%	NA	NA	NA	4
	51, M	E200K	NA		NA	NA	NA	10
	56, M	E200K	NA		NA	NA	NA	74
	49, M	E200K	NA		NA	NA	NA	17
	41, F	E200K	NA		NA	NA	NA	15
	51, M	E200K	NA		NA	NA	NA	97
	53, M	E200K	NA		NA	NA	NA	4
	42, M	E200K	NA		NA	NA	NA	4
	40, F	E200K	NA		NA	NA	NA	2
	49, M	E200K	NA		NA	NA	NA	30
Hassan et al, 2021, UK	61, M	E200D	M/M	Cognitive dysfunction, gait disturbance	Basal ganglia, cerebral cortex	-	-	3
Present patient, Chinese	47, F	T188K	M/M	Diplopia, dizziness, gait disturbance	Basal ganglia, cerebral cortex, cerebellar cortex	-	-	15

*PRNP*, prion protein gene; DWI, diffusion-weighted imaging; PSWCs, periodic sharp wave complexes; EEG, electroencephalography; CSF, cerebrospinal fluid; NA, not available.

## Discussion

We describe the first known gCJD patient with a *PRNP* T188K homozygous mutation. T188K gCJD is the second most common observed genetic prion disease in China after fatal familial insomnia caused by the *PRNP* D178N mutation but is rarely reported in other countries [4]. To date, more than 30 gCJD cases with the *PRNP* T188K mutation has been identified, all of which were heterozygous. In 2017, the clinical and laboratory features of T188K heterozygous mutations were summarized by Shi *et al.* [5]. In their series, rapidly progressive dementia and cerebellum symptoms were observed initially in 66.7% and 40% of patients, respectively. Additionally, 83.3% of patients showed restricted diffusion on MRI, 72.3% were positive for CSF 14-3-3 protein and only 17.2% revealed PSWCs on EEG. The median onset age was 59 years old (range: 40–85 years old), and the median duration was 4 months (range: 2–12 months). The clinical manifestations of our patient were mostly consistent with those of patients with T188K heterozygous mutations. However, she had an earlier onset age (47 years old) and a longer survival time (15 months).

The influence of homozygous *PRNP* mutations on the phenotypes of gCJD is unclear. As shown in Table 1, V203I, E200K and E200D are the only three *PRNP* mutations reported as homozygous in gCJD to date [6–8]. Although the clinical features of patients with these homozygous mutations were similar to those of patients with heterozygous mutations, some details should be noted. The V203I homozygous mutation patient had a longer survival time than V203I heterozygous mutation patients [6]. The E200K homozygous mutation patients presented with a younger disease onset and had a longer survival time than E200K heterozygous mutation patients [7]. Clinical features of the E200D homozygous mutation patient were incomparable as E200D heterozygous mutations are highly likely to be benign [8].

With the dysfunction of both alleles, clinical phenotypes of autosomal dominant disorders involving homozygous mutations are expected to be more severe than those involving heterozygous mutations. This has been demonstrated in several diseases, including familial hypercholesterolemia, certain subtypes of spinocerebellar ataxia and dentatorubro-pallidoluysian atrophy [9–11]. However, combining previously reported findings and our current observations, it seems likely that gCJD patients with homozygous *PRNP* mutations have a younger onset age but a longer disease duration than heterozygous patients. The mechanism underlying this phenomenon is unclear but may be associated with the quantities and ratios of cellular PrP (PrP^C^) and PrP^sc^. A high quantity of PrP^sc^ in homozygous patients might increase the chance of spontaneous aggregation and initiate the disease earlier, while the lack of PrP^C^ might slow down the progression of prion diseases and lead to a longer duration [12]. Alternatively, the disease duration might be modified by the age of onset. For example, Boesenberg *et al*. reported that young patients with CJD had significantly longer disease duration than older patients [13]. Further studies are needed to clarify the influence of homozygous mutations on the gCJD phenotype.
